# Utility of Washington Early Recognition Center Self-Report Screening Questionnaires in the Assessment of Patients with Schizophrenia and Bipolar Disorder

**DOI:** 10.3389/fpsyt.2016.00149

**Published:** 2016-08-26

**Authors:** Christina J. Hsieh, Douglass Godwin, Daniel Mamah

**Affiliations:** ^1^Saint Louis University School of Medicine, St. Louis, MO, USA; ^2^Department of Psychiatry, Washington University Medical School, St. Louis, MO, USA

**Keywords:** bipolar disorder, schizophrenia, WERCAP, psychosis, stress, questionnaire

## Abstract

Early identification and treatment are associated with improved outcomes in bipolar disorder (BPD) and schizophrenia (SCZ). Screening for the presence of these disorders usually involves time-intensive interviews that may not be practical in settings where mental health providers are limited. Thus, individuals at earlier stages of illness are often not identified. The Washington Early Recognition Center Affectivity and Psychosis (WERCAP) screen is a self-report questionnaire originally developed to identify clinical risk for developing bipolar or psychotic disorders. The goal of the current study was to investigate the utility of the WERCAP Screen and two complementary questionnaires, the WERC Stress Screen and the WERC Substance Screen, in identifying individuals with established SCZ or BPD. Participants consisted of 35 BPD and 34 SCZ patients, as well as 32 controls (CON), aged 18–30 years. Univariate analyses were used to test for score differences between groups. Logistic regression and receiver operating characteristic (ROC) curves were used to identify diagnostic predictors. Significant group differences were found for the psychosis section of the WERCAP (pWERCAP; *p* < 0.001), affective section of the WERCAP (aWERCAP; *p* = 0.001), and stress severity (*p* = 0.027). No significant group differences were found in the rates of substance use as measured by the WERC Substance Screen (*p* = 0.267). Only the aWERCAP and pWERCAP scores were useful predictors of diagnostic category. ROC curve analysis showed the optimal cut point on the aWERCAP to identify BPD among our participant groups was a score of >20 [area under the curve (AUC): 0.87; sensitivity: 0.91; specificity: 0.71], while that for the pWERCAP to identify SCZ was a score of >13 (AUC: 0.89; sensitivity: 0.88; specificity: 0.82). These results indicate that the WERCAP Screen may be useful in screening individuals for BPD and SCZ and that identifying stress and substance-use severity can be rapidly done using self-report questionnaires. Larger studies in undiagnosed individuals will be needed to test the WERCAP Screen’s ability to identify mania or psychosis in the community.

## Introduction

Schizophrenia (SCZ) and bipolar disorder (BPD) are complex mental illnesses with onset in adolescence and are among the leading causes of disability worldwide (1). Early identification and initiation of antipsychotic therapy are associated with improved prognoses in SCZ, whereas longer durations of untreated illness are associated with worse outcomes (2, 3). This holds true across different nations and cultures (4, 5). Similarly, in BPD, delayed diagnosis and treatment are associated with more hospitalizations, increased suicidality (6), and impaired social function (7). Patients with recurrent depressive episodes in BPD may be misdiagnosed with unipolar depression, leading to a longer duration of untreated illness and worse outcomes (8–10). Misdiagnosis and inappropriate treatment with antidepressants can increase mania and exacerbation of the episode (11). In some practices, it takes an average of about 10 years between the onset of BPD and initiation of treatment with mood stabilizers (12). The challenges involved in early recognition and treatment of SCZ and BPD support the need for a reliable screening instrument.

Bipolar disorder and SCZ are typically diagnosed by a clinician or by structured clinical interviews, which are time consuming and, therefore, not practical for screening purposes. Commonly used screening questionnaires in BPD, such as the Altman Self-Rating Mania Scale (13), Young Mania Rating Scale (14), or the Bipolar Spectrum Diagnostic Scale (BSDS) (15), either probe for severity of current manic-like symptoms or generate unacceptably high rates of false positives and low sensitivity (16). For example, the Mood Disorders Questionnaire, the most extensively studied of instruments for detecting BPD, was found to have a positive predictive value (PPV) of 22.1% (17). Likewise, the BSDS has a PPV of only 16% (18). Thus, the need for a rapid and reliable means to screen for BPD remains unmet. Similarly, the community assessment of psychic experiences (CAPE)-42, a screening instrument for detecting psychosis, has a PPV of only 23.5% (19). Other rating instruments commonly used in assessing SCZ or other psychotic disorders, such as the Scale for the Assessment of Positive Symptoms (20) or the Positive and Negative Syndrome Scale (21), probe for current psychotic symptoms and are not designed to assist in diagnosis.

The Washington Early Recognition Center Affectivity and Psychosis (WERCAP) screen was developed to accurately and efficiently identify individuals at risk of bipolar and psychotic disorders. The WERCAP Screen is a quantitative measure of affective and psychotic symptoms that elicits information about symptom frequency and the degree of resulting dysfunction (“functionality”). The WERCAP Screen has been validated in a non-clinical youth population in the US, provides results comparable to the structured interview for prodromal syndromes (SIPS) in a much shorter period of time, and is designed to be cross-culturally applicable (22). The WERCAP Screen has two sections, the aWERCAP and pWERCAP, which measure the risk of developing BPD (“affectivity”) and psychosis, respectively. Unlike other screening instruments, the WERCAP Screen was designed to assess chronic or lifetime affective or psychotic symptoms (although a time interval can be specified for more acute symptoms). Thus, it could potentially be used to identify individuals with established bipolar or psychotic disorder diagnoses and also inform on illness severity.

Stress (23) and substance use (24) are both associated with risk of psychosis and decompensation in those with psychotic or BPDs; thus, reliably assessing their severities could provide valuable information for clinicians. A number of substances have been found to be associated with risk of psychosis and BPD, most commonly tobacco (25, 26) and cannabis (27). There is now strong evidence suggesting that cannabis use can trigger the onset of psychosis (28–30). Progression to greater cannabis usage frequency is associated with greater risk of psychosis (31). Higher rates of cannabis use have been consistently linked with earlier age of onset in psychosis (30, 32–35) with some studies reporting a similar association in BPD (36–38).

Substance use has also been reported to exacerbate symptoms in patients that have already been diagnosed with SCZ and BPD. SCZ patients with a history of cannabis use tend to have longer hospital stays and more frequent readmissions compared to cannabis non-users (39). Cannabis use is also linked with an increase in psychotic (40–43) and manic symptoms (44). Cannabis use is associated with worse treatment outcomes and functioning in patients with psychosis (45–47) and BPD (48, 49). Some studies have also found tobacco use to be associated with greater illness severity (50). Tobacco smoking is highly prevalent among SCZ and bipolar patients (51) and has been associated with worse outcomes and lower remission rates in BPD (49, 52). In addition, there have been case reports of psychiatric relapse following excessive caffeine consumption in patients suffering from SCZ (53) and BPD (54).

Psychosocial stress is another risk factor shared between SCZ and BPD, along with other psychiatric disorders (55, 56). There is extensive evidence linking early childhood trauma with increased risk of psychosis (57–59) and BPD (60). It has been hypothesized that childhood adversity results in increased vulnerability to stress and psychiatric disorder (61). SCZ, BPD, and depression patients all tend to report increased sensitivity to stress (62). Moreover, greater exposure to childhood adversity has been correlated with higher levels of perceived stress and increased psychopathology (63). Stressors later in life have also been found to increase the risk of psychotic (64) and manic (65–67) episodes. More recent studies have reported everyday stressors in adult life to be correlated with an increase in psychotic (68) and manic (69) symptoms. Thus, assessing stress severity could conceivably improve the identification of individuals at risk for psychosis and BPD.

We have previously developed tools to capture psychosocial stress load and substance-use habits. The WERC Stress Screen (hereafter referred to as “Stress Screen”) is a self-report questionnaire that assesses the overall stress burden currently experienced by the individual, and also quantifies the individual contributions of common psychosocial stressors to the total stress burden (22). The WERC Substance Screen (hereafter referred to as “Substance Screen”) allows respondents to self-report on the usage frequencies of multiple substances that may influence brain function.

The current study explores the utility of our quantitative screening instruments in a sample of patients with SCZ and BPD. Our objective was to determine whether the WERCAP Screen, Stress Screen, or Substance Screen could predict diagnosis in a clinical population and the optimal cutoff thresholds on these instruments. We hypothesized that the measures of psychosis and affectivity would be the most useful predictors of diagnosis.

## Materials and Methods

The Institutional Review Board of Washington University Medical School in St. Louis approved all procedures and study materials. All participants in the study provided informed consent.

### Participants

One hundred two individuals ages 18–30 years participated in the study, which consisted of three groups: BPD (BPD, *n* = 35), SCZ (SCZ, *n* = 34), and healthy controls (CON, *n* = 32). Participants were recruited using a combination of flyers posted in public locations, recruitment information posted to university websites, as well as clinic referrals. Consequently, our sample size was limited by clinical availability of individuals with SCZ and BPD who met inclusion criteria. Diagnosis was determined based on the agreement of assessments by a research psychiatrist and a trained research assistant using the structured clinical interview for DSM-IV axis I disorders (SCID-I) and the structured clinical interview for DSM-IV axis II disorders (SCID-II), with precedence given to the psychiatrist’s determination. Exclusion criteria during initial screening included (1) a history of substance dependence or substance abuse during the 6 months prior to participation, (2) a currently unstable clinical disorder or severe general medical disorder, or (3) a history of head injury, multiple seizures, or concussions resulting in unconsciousness for more than 30 min. Table 1 presents demographic information for the three participant groups.

**Table 1:** **Demographics for each diagnostic group**.

	Control (*N* = 32)	Bipolar (*N* = 35)	Schizophrenia (*N* = 34)	Total (*N* = 101)
Mean age (SD)	24.1 (3.03)	25.1 (3.48)	25.8 (3.44)	25.0 (3.37)

**Characteristic**	***N* (%)**	***N* (%)**	***N* (%)**	***N* (%)**

**Gender**
Female	20 (62.5)	25 (71.4)	7 (20.6)	52 (52.5)
Male	12 (37.5)	10 (28.6)	27 (79.4)	49 (48.5)
**Ethnicity**
American Indian/Alaskan native	0 (0.0)	0 (0.0)	0 (0.0)	0 (0.0)
Asian	3 (9.4)	3 (8.6)	0 (0.0)	6 (5.9)
Black/African-American	11 (34.0)	7 (20.0)	21 (62.4)	39 (38.6)
Native Hawaiian/Pacific Islander	0 (0.0)	0 (0.0)	0 (0.0)	0 (0.0)
White	17 (53.1)	24 (68.6)	11 (31.4)	52 (51.0)
Biracial/Multiracial	1 (3.1)	1 (2.9)	1 (2.9)	3 (3.0)
Other/unknown	0 (0.0)	0 (0.0)	1 (2.9)	1 (1.0)
**Employment**				
Full time	15 (46.9)	12 (34.3)	1 (2.9)	28 (27.7)
Part time	6 (18.8)	6 (17.1)	7 (20.0)	19 (18.8)
Unemployed	2 (6.3)	8 (22.9)	21 (61.8)	31 (30.7)
Full-time student	6 (18.8)	5 (14.3)	4 (11.4)	15 (14.9)
Part-time student	3 (9.4)	3 (8.6)	0 (0.0)	6 (5.9)
Other	0 (0.0)	1 (2.9)	0 (0.0)	1 (1.0)
**Level of education**
Graduate professional training	5 (15.6)	4 (11.4)	0 (0.0)	9 (8.9)
College/university graduate	10 (31.3)	7 (20.0)	1 (2.9)	18 (17.8)
Partial college	14 (43.8)	18 (51.4)	15 (44.1)	47 (46.5)
High school graduate	0 (0.0)	5 (14.3)	13 (38.2)	18 (17.8)
Partial high school	3 (9.4)	1 (2.9)	3 (8.8)	7 (6.9)
Junior high school	0 (0.0)	0 (0.0)	1 (2.9)	1 (1.0)
Less than 7 years of school	0 (0.0)	0 (0.0)	0 (0.0)	0 (0.0)
Unknown	0 (0.0)	0 (0.0)	0 (0.0)	0 (0.0)

### WERC Screening Forms

Participants completed three screening forms: the WERCAP Screen, the Stress Screen (22), and the Substance Screen. These forms are available for public use (http://werc.wustl.edu/home/screeninginstruments).

The WERCAP Screen assesses risk of BPD and psychosis based on a quantification of lifetime symptom burden. The WERCAP Screen consists of a total of 16 questions, the first half of which are designed to assess affective symptoms experienced by individuals, while the remaining questions assess risk of psychosis. The majority of questions (10 out of 16) require two responses, including a rating of the frequency of symptom occurrence, and if present, the severity of the associated functional impairment. For the item probing into decreased need for sleep, respondents were asked to rate symptom duration rather than degree of functional impairment. Six questions in the affectivity section assess only symptom frequency, since they inquire about symptoms that do not typically impair function, or the degree of resulting dysfunction is difficult to assess. The responses were converted into numerical values as follows: No = 0, Once = 1, Rarely (<yearly) = 2, Sometimes (>yearly–monthly) = 3, Often (>monthly–weekly) = 4, and Almost Always (>weekly–daily) = 5. For items assessing effect on functionality, responses were converted as follows: Not at All = 0, A Little = 1, Moderately = 2, Severely = 3. The frequency and functionality scores for items 1–8 were summed to generate a composite aWERCAP (affectivity) score, and the remaining items (9–16) were summed to generate the composite pWERCAP (psychosis) score. The maximum aWERCAP score possible is 49, and the maximum pWERCAP score is 64, yielding a maximum total WERCAP Screen score of 113.

The Stress Screen assesses the total stress burden of 23 common psychosocial stressors, such as one’s relationships with family and friends, substance use, or the workplace [see Ref. (22) for a complete list]. Space is also provided to write in up to two additional stressors. By default, the Stress Screen is designed to capture the current stress load on an individual. Respondents are asked to rate the extent to which they are affected by each stressor by marking the appropriate checkbox. Each response is converted to a numerical rating (No = 0, A Little = 1, Moderate = 2, A Lot = 5, Severely = 10), and then summed to generate the Stress Screen score. The maximum Stress Screen score possible is 230.

The Substance Screen is a 20-item questionnaire that assesses the current substance-use habits of the respondent. Respondents were asked to rate their usage frequency of a variety of psychotropic substances, such as caffeine, nicotine, prescription drugs, etc. [see Ref. (22) for a complete list] and could also choose to write in up to two additional substances. The frequency responses were converted into a numerical score using the same scale as that in the WERCAP Screen, and then summed to generate the Substance Screen score.

### Data Analysis

Statistical analyses were performed using IBM’s SPSS software (IBM SPSS Statistics for Macintosh, Version 24.0, Armonk, NY, USA). Demographic differences between diagnostic groups were assessed with one-way ANOVAs treating diagnosis as a fixed factor or chi-square (χ^2^) tests as appropriate. Between-group differences in aWERCAP, pWERCAP, and Stress Screen scores were examined using univariate analysis of covariance (ANCOVA) tests, including gender, age, and years of education as covariates, followed by *post hoc* pairwise tests, corrected for multiple comparisons treating diagnostic group as a fixed effect.

We utilized logistic regression to examine the capability of the aWERCAP, pWERCAP, WERC Stress Screen, and number of substances used to predict diagnostic group. The number of substances measured by the Substance Screen was used in the logistic regression instead of a Substance Screen score derived from summing all substance usage frequencies, due to substantial potency variability of the different substances. Only the most prevalent substances used by our participants were included in the logistic regression (i.e., tobacco, coffee, other caffeinated beverages, alcohol, and cannabis). Receiver Operating Characteristic (ROC) curves were generated for aWERCAP and pWERCAP to study classification with varying thresholds and determine the thresholds with optimum sensitivity and specificity. Substance and Stress Screen scores were not predictive of diagnostic group membership in the logistic regressions and, therefore, were not considered for this analysis.

## Results

### Demographics

Demographic information for each diagnostic group is presented in Table 1. Mean age was not significantly different between groups (*F*_(2,98)_ = 1.848, *p* = 0.163). However, there were significant differences in gender (χ^2^ = 20.122, *p* < 0.001) and ethnicity (χ^2^ = 17.465, *p* = 0.026) between groups. Psychiatric comorbidities in each group are shown in Table 2.

**Table 2:** **Prevalence of psychiatric disorders for each diagnostic group (*N* = 101)**.

	Control (*N* = 32)	Bipolar (*N* = 35)	Schizophrenia (*N* = 34)	Total (*N* = 101)

Psychiatric diagnosis	*N* (%)	*N* (%)	*N* (%)	*N* (%)
No diagnosis	30 (93.8)	1 (2.9)	0 (0)	31 (30.7)
*Mood disorders*	2 (6.25)	33 (94.3)	0 (0)	35 (34.7)
Major depressive disorder	2 (6.25)	0 (0)	0 (0)	2 (2.0)
Bipolar disorder	0 (0)	33 (94.3)	0 (0)	33 (32.7)
*Anxiety disorders*	0 (0)	30 (85.7)	19 (55.9)	49 (48.5)
Generalized anxiety disorder	0 (0)	3 (8.6)	5 (14.7)	8 (7.9)
Panic disorder	0 (0)	2 (5.7)	1 (2.9)	3 (3.0)
Panic disorder w/agoraphobia	0 (0)	0 (0)	3 (8.8)	3 (3.0)
Agoraphobia w/o panic disorder	0 (0)	4 (11.4)	5 (14.7)	9 (8.9)
PTSD	0 (0)	7 (20.0)	4 (11.8)	11 (10.9)
Social phobia	0 (0)	10 (28.6)	2 (5.9)	12 (11.9)
Specific phobia	0 (0)	4 (11.4)	2 (5.9)	6 (5.9)
*Obsessive compulsive disorders*	0 (0)	0 (0)	1 (2.9)	1 (1.0)
Obsessive compulsive disorder	0 (0)	0 (0)	1 (2.9)	1 (1.0)
Body dysmorphic disorder	0 (0)	0 (0)	0 (0)	4 (4.0)
*Personality disorders*	0 (0)	22 (62.9)	9 (26.5)	31 (30.7)
Borderline PD	0 (0)	5 (14.3)	2 (5.9)	7 (6.9)
Avoidant PD	0 (0)	8 (22.9)	4 (11.8)	12 (11.9)
Schizotypal PD	0 (0)	0 (0)	1 (2.9)	1 (1.0)
Obsessive compulsive PD	0 (0)	5 (14.3)	1 (2.9)	6 (5.9)
Antisocial PD	0 (0)	1 (2.9)	0 (0)	1 (1.0)
Dependent PD	0 (0)	3 (8.6)	1 (2.9)	4 (4.0)
Paranoid PD	0 (0)	5 (14.3)	4 (11.8)	9 (8.9)
Narcissistic PD	0 (0)	3 (8.6)	1 (2.9)	4 (4.0)
*Psychotic disorders*	0 (0)	1 (2.9)	34 (100.0)	35 (34.7)
Schizoaffective disorder	0 (0)	1 (2.9)	4 (11.8)	5 (5.0)
Schizophrenia	0 (0)	0 (0)	29 (85.3)	29 (28.7)
Psychosis NOS	0 (0)	0 (0)	1 (2.9)	1 (1.0)
*Substance-related disorders*	0 (0)	0 (0)	2 (5.9)	2 (2.0)
Cannabis abuse	0 (0)	1 (2.9)	2 (5.9)	3 (3.0)
Cannabis dependence	0 (0)	0 (0)	0 (0)	0 (0.0)
Alcohol abuse	0 (0)	0 (0)	0 (0)	0 (0.0)
Alcohol dependence	0 (0)	0 (0)	0 (0)	0 (0.0)
Opioid dependence	0 (0)	0 (0)	0 (0)	0 (0.0)
Polysubstance abuse	0 (0)	0 (0)	0 (0)	0 (0.0)
Hallucinogen dependence	0 (0)	0 (0)	0 (0)	0 (0.0)
Stimulant dependence	0 (0)	0 (0)	0 (0)	0 (0.0)
*Eating disorders*	0 (0)	2 (5.7)	0 (0)	2 (2.0)
Bulimia nervosa	0 (0)	1 (2.9)	0 (0)	1 (1.0)
Binge eating disorder	0 (0)	1 (2.9)	0 (0)	1 (1.0)

### WERCAP Screen

**Figure 1:**
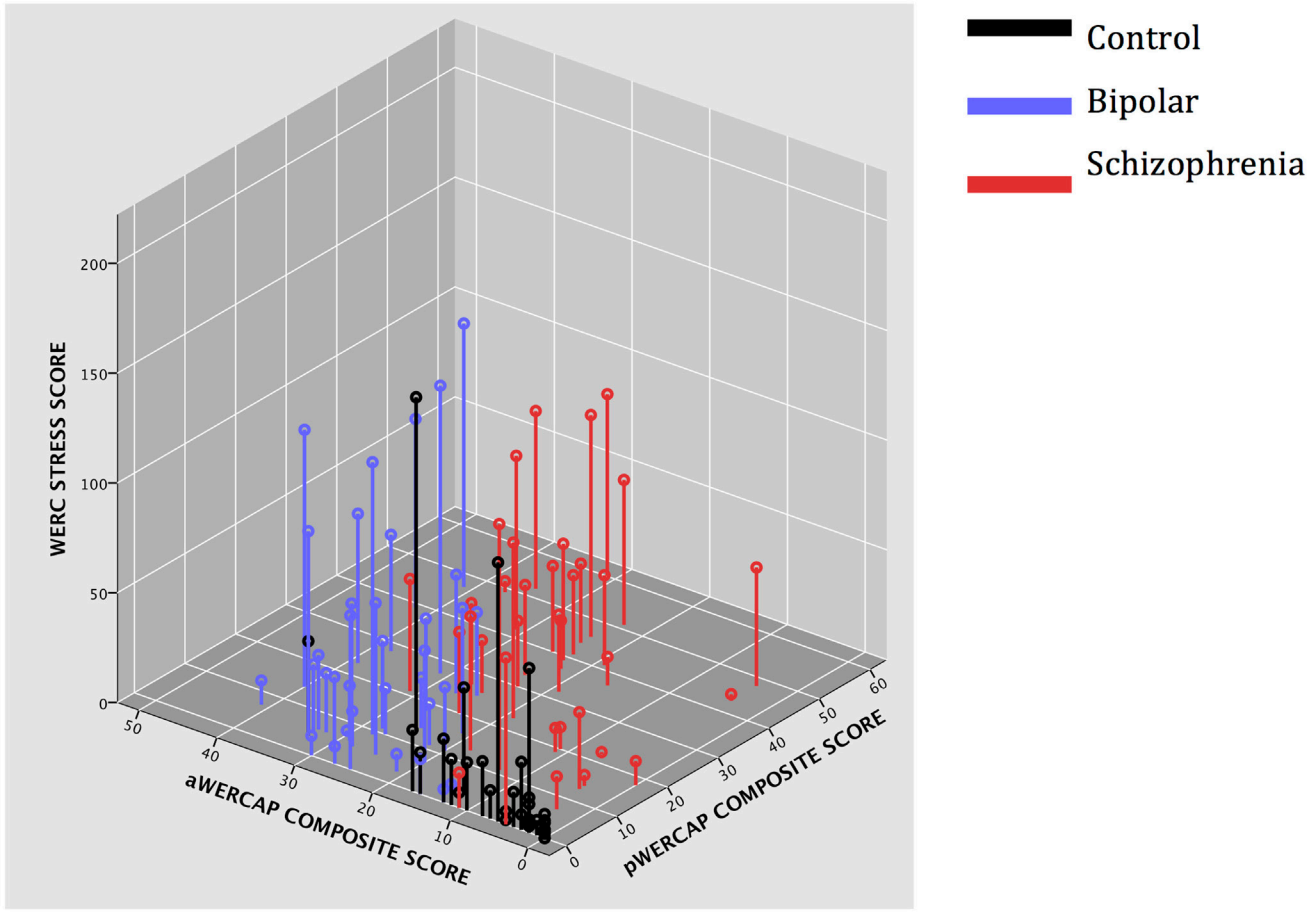
**3D scatter-plot showing Stress Screen scores, aWERCAP composite scores, and pWERCAP composite scores in each diagnostic group**.

**Figure 2:**
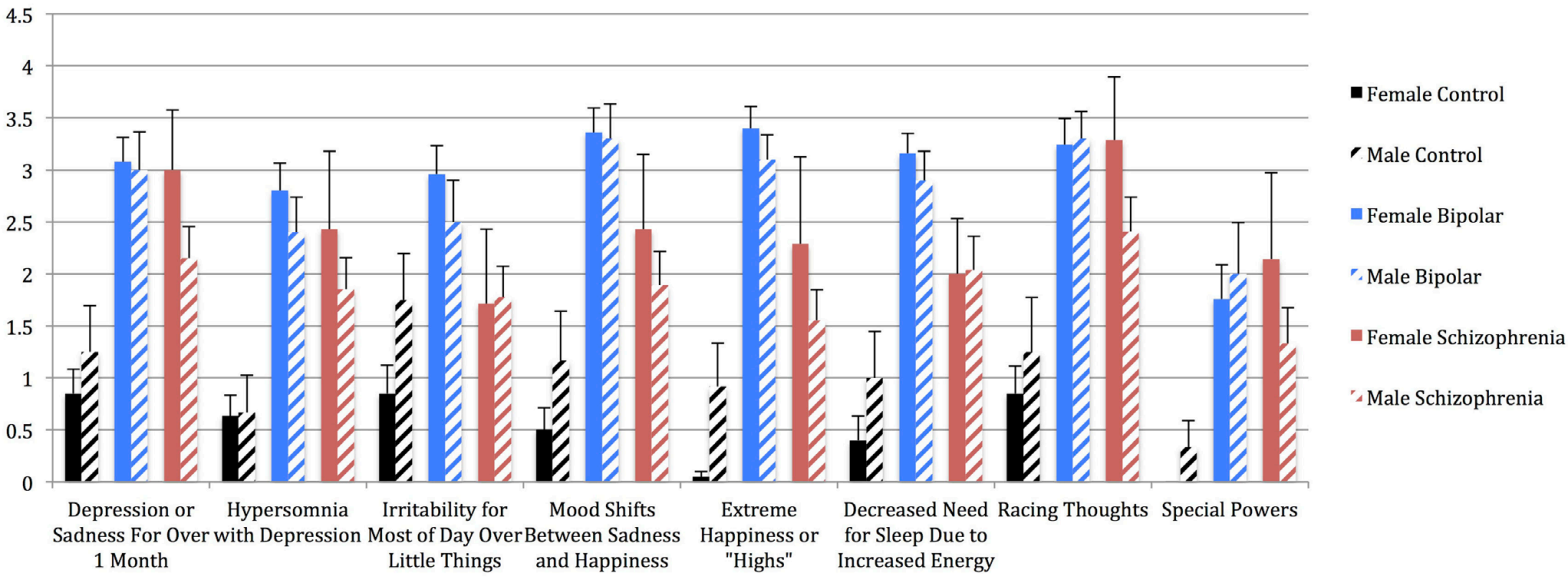
**Mean aWERCAP Screen individual item scores (frequency) in males vs. females of each diagnostic group, with standard error bars**. *x*-axis = individual aWERCAP items, *y*-axis = mean aWERCAP frequency score, derived by summing and averaging the frequency responses in the affectivity section.

**Figure 3:**
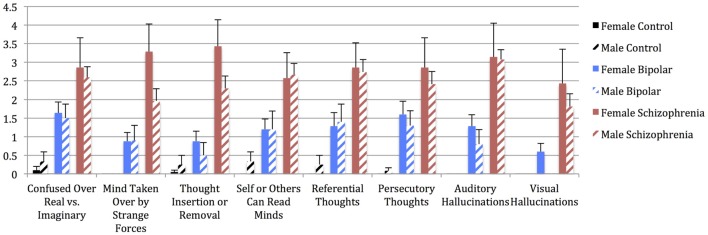
**Mean pWERCAP Screen individual item scores (frequency) in males vs. females of each diagnostic group, with standard error bars**. *x*-axis = individual pWERCAP items, *y*-axis = mean pWERCAP frequency score, derived by summing and averaging the frequency responses in the psychosis section.

Univariate analyses showed significant group effects for aWERCAP (*F*_(2,92)_ = 52.904, *p* < 0.001) and pWERCAP (*F*_(2,92)_ = 50.378, *p* < 0.001, η^2^ = 0.523), but not for stress or substance scores. The relationship between individual aWERCAP, pWERCAP, and Stress Screen scores is depicted in Figure 1. Average scores for each of the screens are shown in Table 3. Pairwise comparisons between diagnoses, Bonferroni correcting for multiple comparisons, showed that BPD had significantly higher aWERCAP scores than CON (*p* < 0.001) and SCZ (*p* = 0.001). Scores on the aWERCAP in SCZ were also higher than in CON (*p* < 0.001). pWERCAP scores were significantly higher in SCZ compared to BPD (*p* < 0.001) and CON (*p* < 0.001), as well as in BPD compared to CON (*p* = 0.001). Mean aWERCAP scores did not significantly differ between genders (*t* = 0.691, *p* = 0.491). Mean aWERCAP item scores broken down by sex and diagnosis are shown in Figure 2. Males experienced significantly higher total psychotic symptom scores than females (*t* = −2.324, *p* = 0.022). Mean pWERCAP item scores for males and females in each diagnostic group are shown in Figure 3.

**Table 3:** **Mean (SD) WERC Screen scores and number of substances used in each diagnostic category**.

	Control	Bipolar	Schizophrenia
Stress Screen	24 (34)	46 (37)	39 (28)
Number of substances	3.3 (0.4)	4.7 (0.4)	3.4 (0.4)
aWERCAP	7.1 (8.1)	29.2 (7.8)	19.0 (11.0)
pWERCAP	0.8 (3.1)	13.2 (12.3)	31.8 (16.8)

### Stress Screen

**Figure 4:**
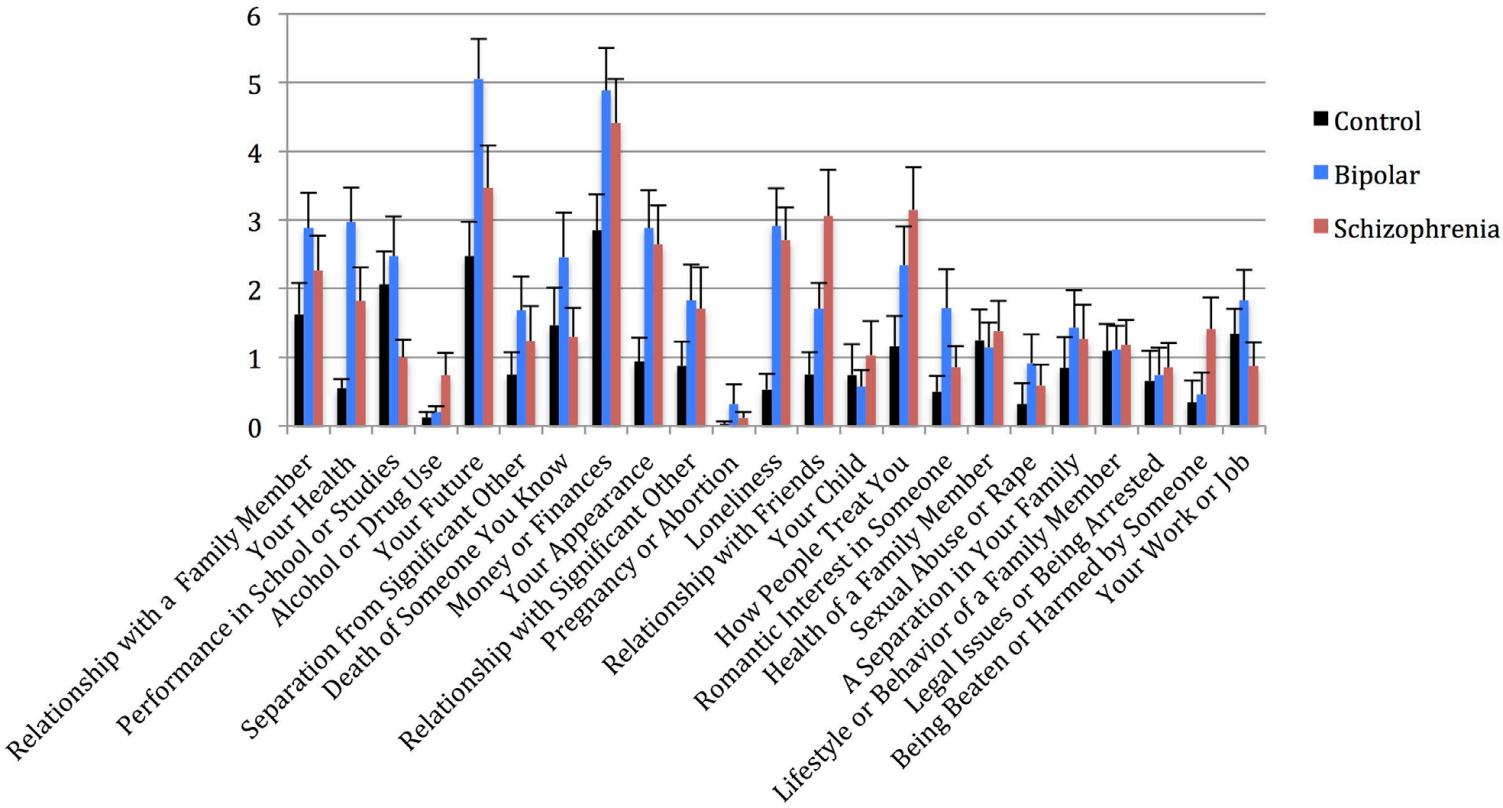
**Mean WERCAP Stress Screen individual item scores in each diagnostic group, with standard error bars**. *x*-axis = individual Stress Screen items, *y*-axis = mean Stress Screen scores for each item.

Univariate analysis of Stress Screen scores showed a significant main effect of diagnostic group (*F*_(2,92)_ = 3.740, *p* = 0.027). Mean Stress Screen item scores for each diagnostic group are shown in Figure 4. Pairwise comparisons showed that BPD had significantly higher Stress Screen scores than the CON (*p* = 0.033). However, BPD did not significantly differ from the SCZ on stress severity. SCZ did not significantly differ from CON on stress severity (*p* = 0.182). We found no significant differences in WERC Stress scores across genders (*t* = 0.541, *p* = 0.589).

### Substance Screen

A comparison of the rate of substance use showed only a marginal effect of diagnosis (*F*_(2,92)_ = 2.987, *p* = 0.055). BPD reported using significantly more substances than CON (*p* = 0.015) or SCZ (*p* = 0.032). There was no significant difference in the number of substances used between the CON and SCZ. SCZ reported tobacco use at significantly higher rates than CON (*p* = 0.021). BPD had significantly higher coffee consumption rates than SCZ (*p* = 0.02). We found no significant differences in the rates of substance use as measured by the WERC Substance Screen (*t* = 1.117, *p* = 0.267).

### Logistic Regression – Predicting Diagnostic Group

Overall, logistic regression models using the aWERCAP, pWERCAP, Stress Screen, and number of substances used as predictors correctly predicted diagnosis in 89.1% of participants. Specifically, the model correctly predicted 93.8% of CON, 85.7% of BPD, and 88.2% of SCZ. However, only the aWERCAP (χ^2^ = 47.835, *p* < 0.001) and pWERCAP (χ^2^ = 79.974, *p* < 0.001) scores were useful predictors of diagnostic category, whereas the Stress Screen (χ^2^ = 2.587, *p* = 0.274) and number of substances used (χ^2^ = 2.918, *p* = 0.232) did not significantly contribute to group prediction. Only the aWERCAP score significantly predicted BPD (*p* < 0.001), and only the pWERCAP score significantly predicted SCZ (*p* = 0.001). Results of the logistic regression are presented in Table 4.

**Table 4:** **Logistic regression analysis of WERC Screens for diagnosis, with control or schizophrenia group as reference category**.

Predictor	β	SE β	Wald’s χ^2^	df	*p*	*e*^β^ (odds ratio)
**Bipolar disorder^a^**						
Constant	−6.446	1.721	14.033	1	<0.001	
aWERCAP	0.279	0.078	12.956	1	<0.001	1.322
pWERCAP	0.282	0.165	2.915	1	0.134	1.326
WERC stress	−0.028	0.018	2.241	1	0.973	0.973
Substances used	0.343	0.222	2.390	1	0.122	1.409
**Schizophrenia^a^**						
Constant	−2.310	1.210	3.647	1	0.056	
aWERCAP	−0.074	0.083	0.802	1	0.370	0.928
pWERCAP	0.520	0.170	9.358	1	0.002	1.682
WERC stress	−0.020	0.020	1.038	1	0.308	0.980
Substances used	0.107	0.281	0.146	1	0.702	1.113
**Bipolar disorder^b^**						
Constant	−4.136	1.683	6.042	1	0.014	
aWERCAP	0.353	0.096	13.521	1	<0.001	1.424
pWERCAP	−0.237	0.063	14.073	1	<0.001	0.789
WERC stress	−0.007	0.013	0.297	1	0.583	0.993
Substances used	0.236	0.246	0.914	1	339	1.266

**Test**			**χ^2^**	**df**	***p***	

Overall model evaluation						
Likelihood ratio test			156.555	8	<0.001	
Goodness-of-fit test						
Pearson			153.307	190	0.976	

*^a^Results of multinomial logistic regression predicting diagnosis. Cox and Snell R^2^ = 0.78, Nagelkerke R^2^ = 0.886, McFadden R^2^ = 0.706. Healthy controls were treated as the reference group in the above analysis*.

*^b^Schizophrenia was treated as the reference category in the above analysis for comparison against the bipolar group*.

### ROC Curves for WERCAP

**Figure 5:**
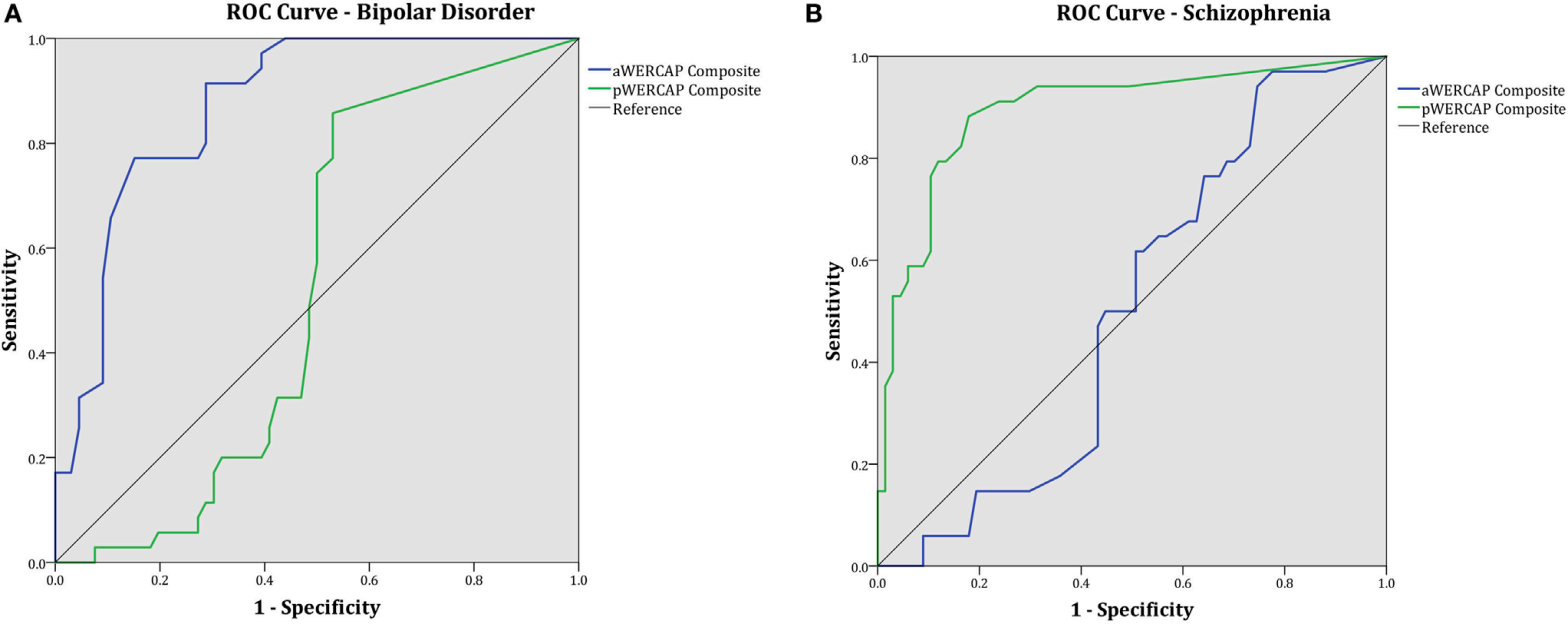
**WERCAP Screen receiver operating characteristic (ROC) curve and area under the curve (AUC) for detecting (A) bipolar disorder and (B) schizophrenia**.

To evaluate the discriminative power of the WERCAP screen to detect BPD and SCZ, ROC curves were calculated for aWERCAP and pWERCAP (see Figure 5). For detecting BPD, aWERCAP performed significantly better than chance, with an area under the curve (AUC) of 0.873 (*p* < 0.001); however, pWERCAP was not significantly better than chance at detecting BPD (AUC = 0.524, *p* = 0.695). The optimal cutoff on the aWERCAP to identify BPD was 20 (Table 5). At this cut point, sensitivity was 0.91 and specificity was 0.71.

**Table 5:** **ROC curve analysis for WERCAP and diagnosis**.

Cutoff	Sensitivity		Specificity		PPV		NPV	
	aWERCAP	pWERCAP	aWERCAP	pWERCAP	aWERCAP	pWERCAP	aWERCAP	pWERCAP
0	100.00	100.00	0.00	0.00	50.0	50.0	n/a	n/a
1	100.00	94.00	14.00	51.00	54.0	65.7	100.0	89.5
2	100.00	94.00	15.00	51.00	54.0	65.7	100.0	89.5
3	100.00	94.00	24.00	51.00	57.0	65.7	100.0	89.5
4	100.00	94.00	29.00	55.00	58.0	67.6	100.0	90.2
5	100.00	94.00	36.00	60.00	61.0	70.1	100.0	90.9
6	100.00	94.00	41.00	64.00	63.0	72.3	100.0	91.4
7	100.00	94.00	42.00	69.00	63.0	75.2	100.0	92.0
8	100.00	91.00	45.00	73.00	65.0	77.1	100.0	89.0
9	100.00	91.00	47.00	73.00	65.0	77.1	100.0	89.0
10	100.00	91.00	47.00	75.00	65.0	78.4	100.0	89.3
11	100.00	91.00	48.00	76.00	66.0	79.1	100.0	89.4
12	100.00	88.00	55.00	82.00	69.0	83.0	100.0	87.2
**13**	100.00	**88.00**	56.00	**82.00**	69.0	**83.0**	100.0	**87.2**
14	97.00	82.00	61.00	84.00	71.0	83.7	95.0	82.4
15	94.00	79.00	61.00	87.00	71.0	85.9	91.0	80.6
16	94.00	79.00	61.00	87.00	71.0	87.0	91.0	81.0
17	91.00	79.00	64.00	88.00	72.0	88.0	88.0	81.00
19	91.00	76.00	67.00	90.00	73.0	88.4	88.0	78.9
**20**	**91.00**	74.00	**71.00**	90.00	**76.0**	88.1	**89.0**	77.6
21	86.00	68.00	71.00	90.0	75.0	87.0	84.0	74.0
22	80.00	65.00	71.00	90.0	73.0	87.0	78.0	72.0
23	77.00	62.00	73.00	90.0	74.0	86.0	76.0	70.0
25	77.00	59.00	85.00	93.00	84.0	89.4	79.0	69.4
26	66.00	59.00	89.00	94.00	86.0	91.0	72.0	70.0
30	40.00	53.00	91.00	97.00	82.0	94.6	60.0	67.4
35	17.00	44.00	97.00	97.00	85.0	93.6	54.0	63.4
40	11.00	35.00	100.00	99.00	100.0	97.2	53.0	60.4
45	0.00	18.00	100.00	99.00	n/a	94.7	50.0	54.7

*Values shown reflect the aWERCAP’s predictive capability of bipolar disorder and the pWERCAP’s ability to predict schizophrenia. Bolded figures indicate values associated with recommended cutoff points*.

For detecting SCZ, pWERCAP demonstrated an AUC of 0.894 (*p* < 0.001), indicating that pWERCAP has very good discrimination for SCZ. aWERCAP was not significantly better than chance at detecting SCZ (AUC = 0.504, *p* = 0.951). The optimal cutoff on the pWERCAP to identify SCZ in this study population was 13 (Table 5). At this cut point, sensitivity was 0.88 and specificity was 0.82.

## Discussion

A goal of this study was to test whether the WERC questionnaires could reliably identify participants with BPD and SCZ. We first investigated performance on these questionnaires by participants with these diagnoses and controls. There was a significant main effect of diagnosis for WERCAP scores, stress severity, and the number of substances used. As we predicted, individuals with BPD scored significantly higher on the affective symptoms (aWERCAP) than the control or SCZ groups. The SCZ group, however, had higher affective symptoms than controls, consistent with the increased affective symptoms (primarily depressive symptoms) often seen in SCZ patients (70–76). Both of our patient groups also had higher psychotic symptoms (pWERCAP) than the control group, with the SCZ patients having the most severe symptoms. The BPD group, however, reported the most significant psychosocial stress severity, as well as the most substances used. This is consistent with results from previous studies showing BPD with a higher prevalence of substance-use disorders (SUDs) than any other psychiatric disorder (77). A high Stress Screen score may not necessarily indicate greater stress reactivity to everyday stressors. Higher sensitivity to stress has been associated with psychosis risk (78–80) and also has been reported in SCZ (81) as well as BPD (82). In addition, several reports have associated childhood adversity with risk of psychosis (58, 83), suggesting the importance of documenting lifetime stressors. Abnormalities in the hypothalamic–pituitary–adrenal axis, a key mediator of the stress response, have been implicated in both SCZ (84) and BPD (85, 86). Stress hypersensitivity and psychosocial stress increase susceptibility to SCZ (87, 88) and BPD (89, 90) and also exacerbate symptoms in both disorders (91). Index manic (69) and psychotic (92) episodes are often preceded by an increase in stressful life events. Thus, the WERCAP Stress Screen may be a useful tool for schools and clinics in monitoring at-risk individuals to prevent onset or relapse.

The affectivity (aWERCAP) score alone identified individuals with BPD in our sample with high sensitivity and specificity, suggesting that the aWERCAP may be useful for detecting BPD in larger settings. Our current findings indicated a minimum aWERCAP cutoff score of 20 for detection of BPD diagnosis, below which an individual would be unlikely to have this diagnosis. Higher scores, however, do not necessarily imply a diagnosis of BPD, as there are likely other conditions that could be associated with severe affective symptoms. While not addressed in the current study, it is plausible, for example, that individuals with certain personality disorders that involve mood lability, such as borderline personality disorder, may report high aWERCAP scores. Depressive disorders may also sometimes be associated with mood lability or irritability, although the majority of the aWERCAP question items would be unlikely to be endorsed by affected individuals. It is, therefore, expected that the aWERCAP alone would result in high false positive rates for BPD in community surveys, and would be insufficient to identify affected individuals. However, the aWERCAP may be suitable as an initial screening tool that would identify individuals who may require clinical or structured assessments.

For the pWERCAP, a cutoff score of 13 was associated with 88% sensitivity, 82% specificity, 83% PPV, and 87% negative predictive value for SCZ diagnosis in our study sample. This score seemed low, considering that in our previous study (22), a score of >30 was found to best correlate with risk for developing a psychotic disorder using a gold standard of psychosis-risk assessment. Thus, a higher score would be expected in SCZ patients. One explanation for this discrepancy is that our study population was largely medicated and stable and, therefore, would exhibit milder symptoms than untreated patients or individuals. Although the WERCAP Screen was intended to elicit chronic or lifetime symptoms, it is plausible that individuals who have been psychiatrically stable could nevertheless underestimate their symptom severity. Thus, we believe that a score of 13 may be too conservative a cutoff in community settings, and would likely identify individuals without any significant psychopathology. Psychotic-like experiences are relatively common, especially at younger ages, and most with such experiences never develop psychotic disorders (93–96). For screening individuals with psychotic disorders in an unmedicated population, a higher cutoff score would, therefore, likely be more specific.

Contrary to our hypothesis, the Stress Screen score and number of common substances (tobacco, coffee, other caffeinated beverages, alcohol, and cannabis) were not significant predictors of diagnostic category, suggesting that these characteristics are non-specific to psychiatric diagnoses. This is not entirely surprising, as multiple other psychiatric disorders, including depression, anxiety, and externalizing disorders have been associated with psychosocial stress (97, 98) and substance use (99, 100). With regard to substance-use assessment, it should be noted that the Substance Screen by default captures current substance-use frequency. It is, thus, conceivable that lifetime exposure to specific substances would be more predictive of BPD or SCZ than recent exposure. Diagnoses of SUDs frequently precede onset of SCZ and BPD (24). Certain substances are specifically associated with symptom onset or exacerbation. For example, cannabis use increases the risk of subsequent mania (101) and psychosis (102). Substance use has also been associated with earlier onset of mania (36, 103) and psychosis (104). A meta-analysis found that cannabis and unspecified substance use significantly increased age of onset of psychosis by 2.70 and 2.00 years, respectively, compared to non-substance-using controls (105). Substance abuse has also been reported to predict conversion to psychosis in a population at high clinical risk (106).

Our study provides an initial investigation of the utility of three recently developed screening instruments in identifying SCZ and BPD. Accurately diagnosing these medical disorders would, however, continue to require clinical or structured assessments, as established diagnostic criteria are complex, often involving multiple symptom categories (e.g., positive, negative, and disorganization symptoms for SCZ), specific duration of symptoms, and diagnostic exclusions (107, 108); thus, screening questionnaires should not be expected to be sufficient for diagnosis. Nevertheless, they can be useful for identifying individuals who may require further assessment. A limitation to our study is that it was conducted using a moderately small-sized sample. Furthermore, the study population was not representative of the community, where a wider range of psychopathologies exist which could make identifying bipolar or psychotic disorders more difficult. Thus, larger studies in diverse settings would be needed to adequately test the psychometric properties of these instruments. Nonetheless, this study demonstrates the potential of the WERC Screen as a screening tool for BPD and SCZ, especially in areas with limited access to a trained psychiatrist, or in settings where conducting a detailed interview is not feasible. Well-validated, self-report instruments could play an increasing role in mental health care in the future. There are relatively few mental health providers, with primary care providers providing a greater proportion of mental health care while faced with increasing time constraints (109). Screening for mental illness could also be useful in schools to identify individuals early when intervention could diminish the burden on the illness and improve functioning. Developing and validating improved screening tools for various aspects of psychopathology are, therefore, highly recommended.

## Author Contributions

DM designed and supervised the study. CH and DG performed analyses. CH, DG, and DM wrote the paper. All authors contributed to discussion of results and contributed to the manuscript at all stages.

## Conflict of Interest Statement

The authors declare that the research was conducted in the absence of any commercial or financial relationships that could be construed as a potential conflict of interest.
